# Reviewed article: Mattos CDCL, Tomazelli J, Girianelli VR. Trends and characteristics of congenital syphilis in children under 1 year of age: a time series analysis, municipality of Rio de Janeiro, 2007-2021. Epidemiol Serv Saude. 2026:35;e20250109

**DOI:** 10.1590/S2237-96222026v35e20250109.b

**Published:** 2026-04-17

**Authors:** Carolina Müller Ferreira

**Affiliations:** 1 Universidade de Pernambuco, Recife, PE, Brazil

## Peer review B

## Questionnaire

Does the manuscript contain new and significant information to justify publication? Yes

Does the Abstract (Summary) clearly and accurately describe the content of the article? No

Is the problem significant and concisely stated? Yes

Are the methods described comprehensively? No

Are the interpretations and conclusions justified by the results? Yes

Is adequate reference made to other work in the field? Yes

## Manuscript Structure

Length of article is: Adequate

Number of tables is: Adequate

Number of figures is: Adequate

Please state any conflict(s) of interest that you have in relation to the review of this paper. None

Interest: Good

Quality: Average

Originality: Average

Overall: Average

Recommendation: Major Revision

## Comments

Referente ao conteúdo, estrutura e padronização, são necessários alguns ajustes conforme descrito abaixo:

- Título e resumo (objetivo): Sugere-se rever o titulo para que fique claro que a sífilis avaliada não é no publico em geral e sim, em um público específico, crianças menores de um ano.

- Palavras-chave: Dê preferência para os descritores principais e por ultimo deixe o descritor associado ao delineamento do estudo. Apresentar primeira letra de cada palavra em maiúscula, separadas por ponto e vírgula.

- Resumo (método): Descreva os métodos com mais detalhes (intervenção ou exposição), incluindo o processo de fonte, coleta e análise de dados, para que outros pesquisadores possam replicar o estudo.

- Resumo (resultados): Apresente resultados mais completos, conclusivos, com linguagem direta mas que seja compreensível. Sugere-se iniciar com total de participantes analisados e sem seguida apresentar os resultados.

- Resumo (conclusão): As conclusões do resumo devem trazer a resposta ao objetivo a partir dos resultados apresentados no resumo. Então sugere-se que corrija a conclusão de modo a apresentar um resumo do achado frente ao objetivo e finalizar com uma frase conclusiva demonstrando a importância/relevância do estudo para ter fácil compreensão. Evite parágrafos circulares, sem conexão com o dados que respondem o objetivo e deixam o texto sem sentido.

- Introdução: São mencionados diversos estudos que deveriam embasar a relevância e diferencial desse trabalho mas ao invés disso, foi escrito com dados soltos e sem um racional ou embasamento da menção dessas informações. Sugere-se que seja rescrito esse penúltimo paragrafo de modo a deixar claro se há estudos similares ou não, e se os estudos realizados até o momento tem limitações (quais), que justificam o porquê esse estudo é necessário.

- Métodos: Os métodos precisam ser descritos com mais detalhes para permitir a replicação do estudo por outros pesquisadores. Descreva o instrumento de coleta de dados e analises estatísticas em detalhes, incluindo informações sobre a validação e confiabilidade das ferramentas usadas. Justifique o tamanho da amostra e explique como os participantes foram selecionados. Inclua um cálculo para demonstrar que a amostra é suficiente para detectar um efeito clinicamente relevante e representativa da população. Descreva os procedimentos empregados no estudo para minimizar vieses em detalhes.

Recomenda-se o uso do guia de redação correto, para cada desenho. No caso de estudos ecológicos, utilizar essa referência como base: "STROBE-GEMA: a STROBE extension for reporting of geographically explicit ecological momentary assessment studies" https://pmc.ncbi.nlm.nih.gov/articles/PMC11170886/

- Discussão: Conjunções de início de frase que pretendem trazer conexão devem ser evitadas, como por exemplo, “Além de”; “No entanto,”; Igualmente etc.

As conclusões devem ser revisadas para garantir que sejam justificadas pelos resultados e respondam diretamente à pergunta de pesquisa.

- Registro do protocolo: erro na escrita da frase "Não se aplica'

- Referência: Sugere-se rever o formato das referências. Assegure que estão no formato Vancouver (sem doi no final):

[ARTIGO] Autor(es). Título. Nome abreviado do periódico. Ano;Volume(Número):Página. (com a página final abreviada [ex.: 123-5] ou página eletrônica [e-page]).

[PÁGINAS] Após o título deve constar [Internet]. Citação e disponibilidade devem ser em inglês, no formato: [cited aaaa Mmm dd]. Available from:

- Figuras: Deve-se adicionar as informações/legendas dos eixos (x e y) para facilitar o entendimento da imagem. A imagem deve ser fácil de ser compreendida isoladamente, sem materiais de suporte, então reveja a clareza da informação que está sendo reportada como figura. Incluir o (n=x) no título da tabela.

- Notas de rodapé: devem ser utilizadas para esclarecer resultado apresentado, identificadas por letras do alfabeto minúsculas e sobrescritas, em ordem sequencial e separadas por ponto e vírgula. Remover fonte e detalhes metodológicos do rodapé das ilustrações (devem constar somente nos métodos). A autoria das tabelas e figuras deve ser dos autores, dispensando tal indicação em nota de rodapé, semelhante à fonte dos dados e demais detalhes metodológicos, que devem constar nos métodos.

- Tabela: Utilizar ponto como separador de milhar (ex. 2.000) e vírgula para frações (ex. 1,5).

